# Cutaneous Mucormycosis in Buffalos in the Brazilian Amazon Biome

**DOI:** 10.3390/ani14091327

**Published:** 2024-04-29

**Authors:** José Diomedes Barbosa, Camila Cordeiro Barbosa, Carlos Eduardo da Silva Ferreira Filho, José Francisco Gimenez Moran, Carlos Magno Chaves Oliveira, Henrique dos Anjos Bomjardim, Paulo Sérgio Chagas da Costa, Marilene de Farias Brito, Milena Carolina Paz, Eryca Ceolin Lamego, Andréia Spanamberg, David Driemeier

**Affiliations:** 1 Instituto de Medicina Veterinária, Universidade Federal do Pará (UFPA), Castanhal 68740-970, Brazil; camilabarbosamedvet@gmail.com (C.C.B.); carloseduardofilho.mv@gmail.com (C.E.d.S.F.F.); jfrangimenez@gmail.com (J.F.G.M.); cmagno@ufpa.br (C.M.C.O.); 2 Faculdade de Medicina Veterinária, Instituto de Estudos do Trópico Úmido da Universidade Federal do Sul e Sudeste do Pará (Unifesspa), Xinguara 68557-335, Brazil; henriquebomjardim@unifesspa.edu.br; 3 Faculdade Antonio Leite FAL/UniBTA, Campus Castanhal 68742-000, Brazil; paulocosta4621@gmail.com; 4 Departamento de Epidemiologia e Saúde Pública (DESP), Instituto de Veterinária, Universidade Federal Rural do Rio de Janeiro (UFRRJ), Seropédica 23890-000, Brazil; mfariasbrito@uol.com.br; 5 Setor de Patologia Veterinária, Faculdade de Veterinária, Universidade Federal do Rio Grande do Sul (UFRGS), Porto Alegre 91540-000, Brazil; mileenapaz@hotmail.com (M.C.P.); erycalamego@gmail.com (E.C.L.); davetpat@ufrgs.br (D.D.); 6 Setor de Micologia, Faculdade de Veterinária, Universidade Federal do Rio Grande do Sul (UFRGS), Porto Alegre 91540-000, Brazil; spanamberg.ad@gmail.com

**Keywords:** Brazil, *Bubalus bubalis*, fungal infection, *Rhizopus* sp., skin diseases

## Abstract

**Simple Summary:**

The state of Pará is the largest producer of buffalo in the Brazilian Amazon biome and has more than 644,000 heads, placing the northern region of Brazil as the holder of the largest buffalo population in the country. Buffalos are rustic animals with high adaptability; they thrive in diverse environments with considerable variations in climate, relief, and temperature; these animals are susceptible to the most diverse diseases, especially skin diseases caused by fungal infection. There are no studies in the literature that report the occurrence of cutaneous mucormycosis in buffalos. Thus, the objective of this study was to report cases of cutaneous mucormycosis in three different buffalo herds in the state of Pará, Brazil.

**Abstract:**

This is the first description of cutaneous mucormycosis in buffalo in the Brazilian Amazon biome. All buffalo showed apathy, inappetence, weight loss, reluctance to move, and prolonged sternal decubitus. Of the four affected animals, two died 15 and 30 days after the appearance of clinical signs. In the initial phase, the skin lesions were rounded areas with dry central regions, sensitive to palpation, with protruding edges and diameters ranging from 8 cm to 15 cm. These areas of necrosis were isolated or coalescing and present mainly on the limbs and sides. In an advanced stage of the disease, there was detachment of the skin from the necrotic areas with extensive wound formation, which sometimes exposed the subcutaneous tissue. The histopathology of the skin showed a multifocal inflammatory infiltrate composed of intact and degenerated eosinophils surrounded by epithelioid macrophages. At the center of these areas was a focally extensive area of epidermal ulceration characterized by intact and degenerated neutrophils, the necrosis of epithelial cells, and the accumulation of fibrin and erythrocytes. The mycological culture was positive for *Rhizopus* sp. The diagnosis of cutaneous dermatitis caused by *Rhizopus* sp. was based on clinical signs, macroscopic and histopathological findings, and the identification of the fungus by mycological and molecular techniques.

## 1. Introduction

Buffalo are rustic animals with high adaptability; they thrive in diverse environments with considerable variations in climate, relief, and temperature; these animals are used in the production of meat, leather, and milk and in traction activities [1]. Thus, buffalo farming has become a reality in Brazil, gradually offering more products to the consumer market. The state of Pará is the largest producer of buffalo in the Brazilian Amazon biome and has more than 644,000 heads, placing the northern region of Brazil as the holder of the largest buffalo population in the country [2].

Ectoparasitosis caused by lice, mites, and ticks is the most common skin disease, causing substantial economic losses in the buffalo hide trade. Other skin diseases of bacterial, viral, and neoplastic origin are less frequent in buffalo.

Mucormycosis is a fungal infection that affects animals and humans. It is caused by fungi of the class zygomycetes, which are subdivided into the following two families: Entomophthoraceae and Mucoraceae. The genera *Rhizopus* sp., *Mucor* sp., and *Absidia* sp. belong to the Mucoraceae family and are most frequently involved in animal diseases [3,4,5,6,7]. The infection caused by these fungi is called mucormycosis, and these agents are considered opportunistic and nondemanding pathogens that can grow in a wide temperature range (25–55 °C). They can be found ubiquitously in vegetation, soil, air, food, and animal excreta [8]. Fungi of the Mucoraceae family exhibit rapid growth, with the production of spores that are transported through the air. Thus, their dissemination may be widespread, but the infection is more dependent on the individual’s immune status [5,6]. The transmission of these species occurs by the inhalation of airborne spores or inoculation through contaminated wounds [9]. Because of the ability of these fungi to invade blood vessels, small hemorrhages, thrombosis, infarction, and necrosis are often associated with them [4,6].

Mucormycosis is occasionally diagnosed in cattle, and it affects the forestomach, particularly the rumen, and omasum, followed by the reticulum and abomasum. Its etiopathogenesis is linked to episodes of rumen acidosis, mastitis, the peripartum period, immunosuppression, and prolonged use of antimicrobials [3,4,7]. However, there are no studies in the literature that report the occurrence of cutaneous mucormycosis in buffalos. The objective of this study was to report cases of cutaneous mucormycosis in three different buffalo herds in the state of Pará, Brazil.

## 2. Materials and Methods

The study consisted of observations made on four buffalo (Buffalo 1–4) diagnosed with mucormycosis. Epidemiological data (age, sex, breed, location of the farms, type of management adopted on the properties, sanitary conditions of the facilities, and the environment in which these animals are found) were obtained during the technical visit. Animals with clinical signs of skin lesions were submitted to general and specific clinical examinations of the integumentary system in accordance with the findings of Dirksen et al. [10]. In Buffalo 1, a blood sample was collected by jugular venipuncture into sterile vacuum tubes with the anticoagulant EDTA (ethylenediamine tetraacetic acid) for a complete blood count. In this animal, a biopsy was performed at the transition between the intact skin and the injured skin after local anesthesia with 4 mL of 2% lidocaine hydrochloride (40 mg). The collected material was fixed in 10% formalin, processed by the Veterinary Pathology Sector of the Federal University of Porto Alegre (UFRGS), and microbiological analysis was performed at the Mycology Laboratory of UFRGS. In addition, skin sections were stained with Grocott–Gomori’s methenamine silver (GMS). The skin samples were seeded on Sabouraud dextrose agar with chloramphenicol and incubated at 25 °C for 7 days.

Molecular diagnosis was made by extracting the DNA from the colonies using the Qiagen DNeasy Plant^®^ Kit (Qiagen, Hilden, Germany), according to the manufacturer’s instructions. Polymerase chain reaction (PCR) was performed using the universal primers ITS1-F and ITS4-R [11]. Amplification was performed in 25 μL of the final volume containing 1 μL of genomic DNA (1–5 ng/μL), 12.5 μL of the Taq PCR Master Mix Kit (Qiagen), and 0.5 μL of each primer (the final concentration of each primer of 0.2 μM). The reaction conditions were as follows: preincubation at 94 °C for 15 min, followed by 35 cycles consisting of initial denaturation at 94 °C for 30 s, annealing at 51 °C for 45 s, extension at 72 °C, and a final extension at 72 °C for 10 min. The PCR product was analyzed by electrophoresis in 2% agarose gel, stained with ethidium bromide, and visualized in a transilluminator under ultraviolet light (Supplementary Materials).

## 3. Results

Four female Murrah buffalos, aged between 6 and 10 years and weighing between 500 and 600 Kg/LW, were treated in this study. The animals came from three farms. Farm 1 was in the municipality of Castanhal (Buffalo 1), Farm 2 was in the municipality of Rondon do Pará (Buffalo 2), and Farm 3 was in the municipality of Mojú (Buffalo 3 and 4), all in the state of Pará, Brazil. In Farm 1 (1/40; 2.5%) and in Farm 2 (1/63; 1.58%), only one animal had skin lesions, and in Farm 3, two animals (2/300; 0.6%) had skin lesions. All farms used the extensive system of rearing with pastures of *Urochloa brizantha* and *Panicum maximum* cv Mombasa grass. Buffaloes 1, 3, and 4 were from dairy herds, and Buffalo 2 belonged to a beef herd. Milking on Farms 1 and 3 was mechanical and performed once a day. In all farms, the animals received a mineral mixture in appropriate troughs and water, either in artificial drinking troughs or natural springs.

The skin lesions appeared in February (Buffalo 1), July (Buffalo 2), and October (Buffalo 3 and 4) 2023. All buffalo showed apathy, inappetence, weight loss, a reluctance to move, and prolonged sternal decubitus. Of the four affected animals, Buffalos 2 and 3 died 15 and 30 days after the appearance of the clinical signs, respectively. Buffaloes 1 and 4 were placed in isolation and improved after 60 days. According to the data shared by the owner, the clinical signs of Buffalo 1 appeared 4 days after a twin birth, during which it was necessary to perform obstetric maneuvers to remove the stillborn calves.

**Figure 1:**
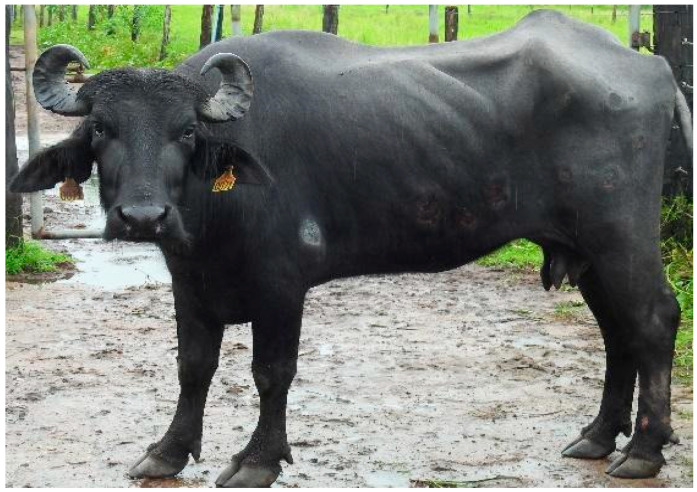
Cutaneous mucormycosis in buffalo in the Brazilian Amazon biome. Buffalo 1: skin lesions characterized by rounded, isolated-to-coalescing areas, with dry and depressed central regions and protruding and irregular borders with varying diameter, Castanhal, Pará state.

**Figure 2:**
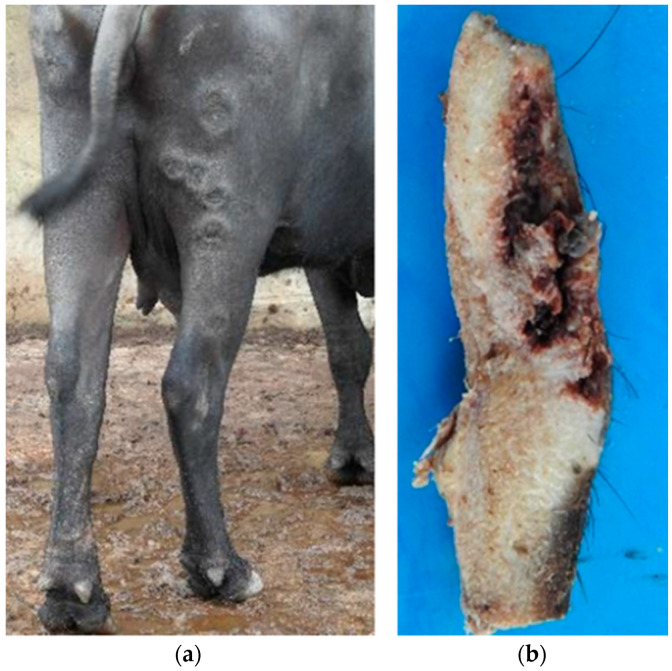
Cutaneous mucormycosis in buffalo in the Brazilian Amazon biome. (**a**) Buffalo from Figure 1, at a closer view, focusing on lesions on the hind limbs; (**b**) cut surface of the skin biopsy of Buffalo 1, performed in the transition area between healthy and lesioned tissues and an area of necrosis involving the epidermis and dermis is shown.

On clinical examination, in the initial phase, the skin lesions (Buffalo 1 and 4) were characterized by rounded areas with dry and depressed central regions, sensitive to palpation, with protruding and irregular borders and a diameter ranging from 8 cm to 15 cm. These areas of necrosis were sometimes isolated and sometimes coalesced and distributed mainly on the limbs and sides (Figure 1 and Figure 2a). 

In the chronic phase of the disease, Buffalos 2 and 3 exhibited the detachment of the skin from the necrotic areas with extensive wound formation (Buffalo 3), which sometimes exposed the subcutaneous tissue. On the cut surface of the skin biopsy of Buffalo 1, which was performed in the transition area between the healthy and injured tissue, there was the detachment of the epidermis around necrosis in the region with small yellowish multifocal areas in the dermis (Figure 2b). Hematologic data from Buffalo 1 showed only moderate anemia.

**Figure 3:**
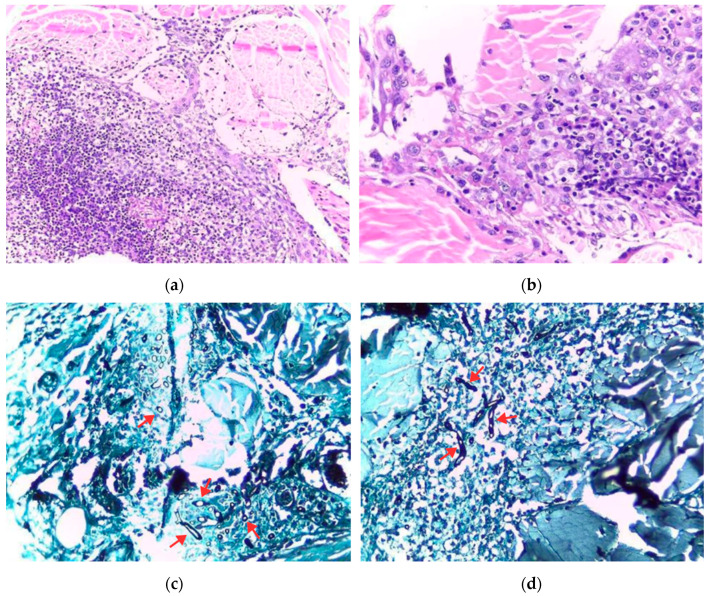
Cutaneous mucormycosis in buffalo in the Brazilian Amazon biome. (**a**,**b**) Histological section of the skin with marked necrosis of the vascular wall and pyogranulomatous inflammatory infiltrate with unstained hyphae. Hematoxylin and eosin staining, obj. 10 and 40; (**c**,**d**) Tubular, irregular in diameter, thick hyphae (arrows) with dichotomous branching and an unstained interior, and a characteristic of the order Mucorales, stained with Grocott–Gomori’s methenamine silver, obj. 20 and 40.

A histological examination of the skin (Buffalo 1) showed the marked multifocal-to-coalescing inflammatory infiltrate of intact and degenerated neutrophils, macrophages, epithelioid macrophages, lymphocytes, and plasma cells. Additionally, there were vasculitis, occasional thrombosis, and transverse and longitudinal sections of fungal hyphae, in addition to the hypertrophy of the vascular wall. In the center of these areas, there were fibers of intensely amorphous eosinophilic material. There was also a focally extensive area of epidermal ulceration characterized by intact and degenerated neutrophils, the necrosis of epithelial cells, and the accumulation of fibrin and erythrocytes (Figure 3a,b). GMS staining of the skin highlighted hyphae, which were tubular, irregular in diameter, and thick, with dichotomous branching and an unstained interior (Figure 3c,d). In addition, the mycological culture was positive for *Rhizopus* sp.

## 4. Discussion

The diagnosis of cutaneous dermatitis caused by *Rhizopus* sp. was based on clinical signs and macroscopic and histological findings, as well as the identification of the fungus by mycological and molecular techniques. The disease affected only female buffaloes over six years of age reared in extensive systems, which might be explained by the presence of few adult male animals in the herds, as two farms followed artificial insemination management.

In cattle, the development of the disease is known to be related to concomitant disturbances or the accidental penetration of the host’s immune defense barriers [4,12]. The stress caused by obstetric maneuvers in Buffalo 1, four days before the onset of the disease, might have triggered the disease in this animal, as reported by Ikeda et al. [3], Jensen et al. [4], and Chihaya et al. [13], who related the occurrence of ringworm to conditions such as long-term treatments with antimicrobials, corticosteroids, peripartum stress, and rumen acidosis.

In the other studied animals raised in extensive systems, heat stress due to the high temperatures in this region caught our attention, associated with the absence of shaded areas for protection during the periods in which the animals were affected. Bernabucci et al. [14] demonstrated that dairy buffalo are more sensitive to heat stress than other buffalo because of higher metabolic heat production, which can negatively affect the intake of dry matter, the time spent standing, and the immune system, with a typical increase in the concentration of plasma cortisol.

Facial subcutaneous destructive mucormycosis has been reported in a three-year-old dog, with the source of cutaneous infection probably being the direct implantation of spores through minor trauma associated with immunosuppression caused by stress [15]. In the present cases, it is possible that the entry point was the skin, unlike the gastrointestinal mucormycosis reported in cattle, in which the mucosa is the main entry point, with a fungal invasion that is usually secondary to other conditions that cause damage to the mucous membranes [7], such as rumenitis due to acidosis.

The severity of the skin lesions varied according to the progression of the disease; in Buffalos 1 and 4, with acute disease, there were only rounded areas with necrosis in the central region of the lesions. According to Choi et al. [6], this is due to the vascular invasion of the agent, which results in thrombosis, infarction, necrosis, and hemorrhage as follows; in Buffaloes 2 and 3, with chronic disease, there was the detachment of the skin from the necrotic areas with wound formation and, sometimes, exposure of the subcutaneous tissue; in Buffalo 1, the histological findings were typical of multifocal-to-coalescing pyogranulomatous inflammation accompanied by vasculitis, occasional thrombosis, and fungal hyphae. These findings are similar to those reported in cattle with mucormycosis [13,16,17], horses [18], and dogs [15,19].

The GMS staining of skin sections was effective in identifying fungal hyphae, as reported in other studies [15,16,19].

The differential diagnosis of the disease was made with traumatic lesions caused by *Mimosa pudica* spines; however, it was discarded because of the location of the lesions, macroscopy, and histopathology, as the lesions caused by the spines of the plant are located mainly in the dorsal region of the distal extremities of the limbs and are ulcerative lesions with irregular contours and serosanguinous exudation [20] unlike the macroscopic characteristics of the skin lesions observed in the buffalo in the aforementioned study. The presence of fungal hyphae shown by the GMS histochemical technique also ruled out traumatic lesions. There was no presence of *M*. *pudica* in the paddocks where the animals grazed.

A PCR, using panfungal (positive) and specific (negative) primers, identified the agent involved and eliminated other probable hyaline fungi. In the present study, the comparative analysis of a nucleotide sequence in the sample showed higher similarity with *Rhizopus* sp. The use of universal primers indicated the genus involved in the case, but specific primers are needed to distinguish between the zygomycetes.

## 5. Conclusions

This is the first report of cutaneous mucormycosis in buffalo in the Brazilian Amazon biome. The onset of this disease was associated with stressful factors such as heat stress and obstructed labor. The macroscopic aspects of the lesion, together with histological changes and molecular techniques, allowed the diagnosis of the disease. Thus, skin diseases are increasingly reported in buffalo species and cause significant economic losses for buffalo farmers in the Brazilian Amazon region.

## Data Availability

The data are contained within the article.

## Supplementary Materials

The following supporting information can be downloaded at: https://www.mdpi.com/article/10.3390/ani14091327/s1, Figure S1: The PCR experiment results after running the gel electrophoresis with 2% agarose gel.
