# Supplementary figures and images for: Cutaneous Mucormycosis in Buffalos in the Brazilian Amazon Biome
Source: Animals (Basel). 2024 Apr 29;14(9):1327. doi: 10.3390/ani14091327 (PMC11083279; doi:10.3390/ani14091327)

**Figure S1:** The PCR experiment results after running the gel electrophoresis with a 2% agarose gel.

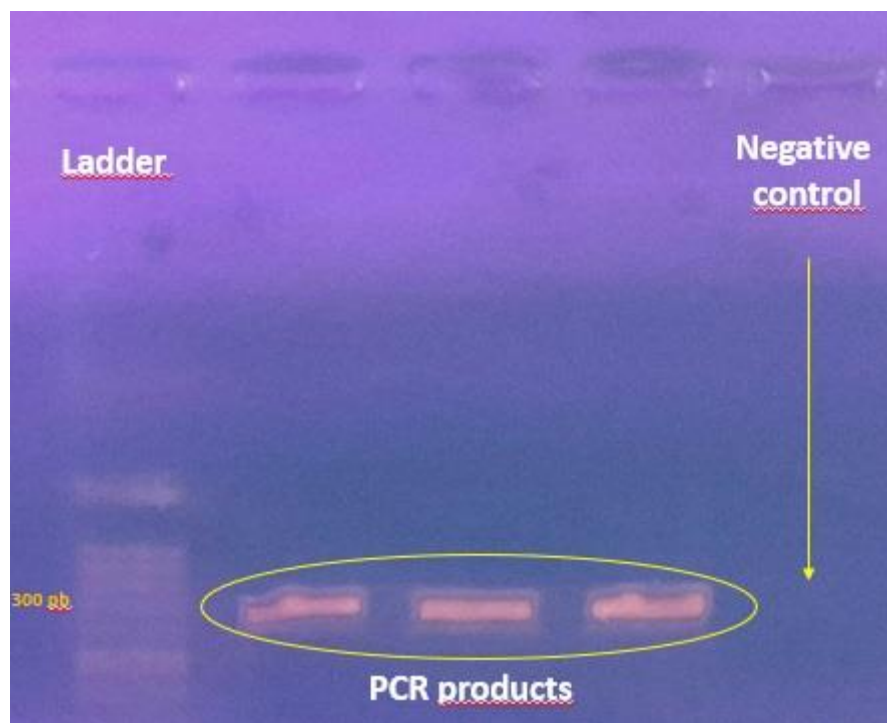

Supplement: Supplementary file 1 [file animals-14-01327-s001.zip › animals-2897850-supplementary.pdf]
